# Subconjunctival and Orbital (Twin) Cysticercosis in a Child

**DOI:** 10.18502/jovr.v14i3.4796

**Published:** 2019-07-18

**Authors:** Kasturi Bhattacharjee, Manpreet Singh, Dipankar Das, Harsha Bhattacharjee

**Affiliations:** ^1^Department of Orbit, Ophthalmic Plastic and Reconstructive Surgery, Sri Sankaradeva Nethralaya, Beltola, Guwahati, Assam, India; ^2^Department of Ophthalmology, Advanced Eye Centre, Post Graduate Institute of Medical Education and Research, Chandigarh, India

##  PRESENTATION

**Figure 1:**
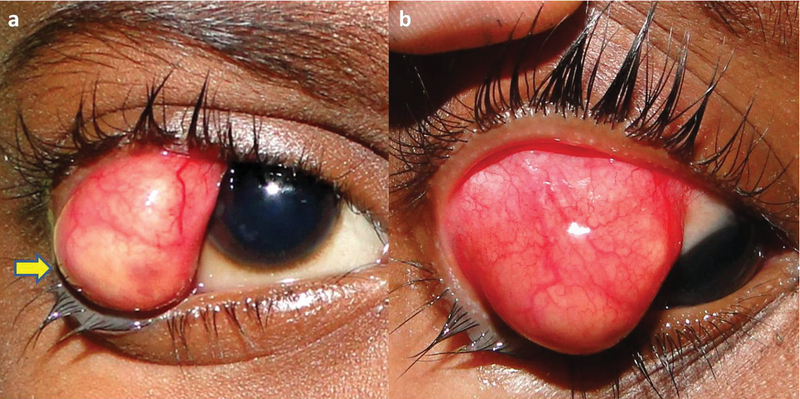
(a) An oval, reddish-yellow cystic mass measuring 15 × 12 mm, protruding from the lateral orbital quadrant and causing the stretching of both tarsal plates and the vertical palpebral fissure. The overlying conjunctiva shows prominent blood vessels. (b) In levodepression, the dilated prominent conjunctival vessels are clearly appreciable on the lateral surface of the cyst.

A seven-year-old Indian boy presented with a reddish mass over the outer part of right eye; the mass had been present for two months. It was associated with pain, discharge, and intermittent double vision. There was no history of ocular trauma, surgery, or any similar complaints. The patient was afebrile with no swelling over any other parts of the body. The ophthalmic examination revealed stretched right upper and lower eyelids, temporal displacement of the lateral canthus, and matting of the eyelashes. A well-defined, reddish yellow, oval mass measuring 15 × 12 mm was noted in the temporal quadrant of the right orbit. The overlying conjunctiva was stretched with prominent blood vessels and a distinctively yellow inferior   segment [Figure1(a), arrow]. Conjunctival congestion was more prominent on the temporal surface of the mass [Figure 1(b)]. A mild restriction of abduction was observed. The rest of the right and left eye examination revealed all findings within normal limits.

**Figure 2:**
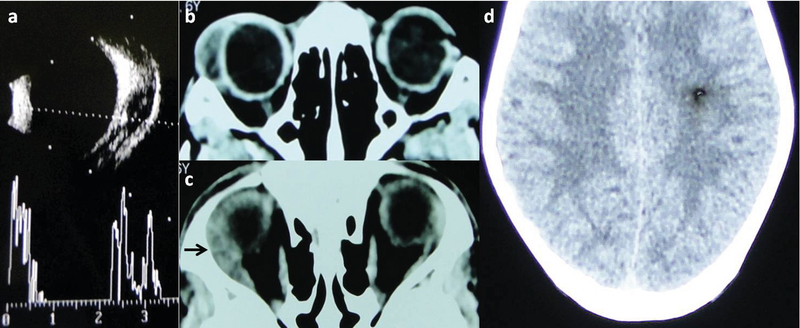
(a) B-scan ultrasound shows a hypoechoic lesion with high internal echogenicity suggestive of scolex in the right lateral rectus muscle. (b) Computed tomography axial scans show a cystic lesion abutting the right globe anteriorly. The heterogenous lesion has a hypodense cavity with a hyperdense internal spec suggestive of a scolex. (c) The superior axial section shows another heterogenous, posterior lesion in the belly of the lateral rectus muscle. (d) The left parietal lobe shows a hypodense lesion with a spec of internal hyper density, even though the classic ring-enhancement is absent.

**Figure 3:**
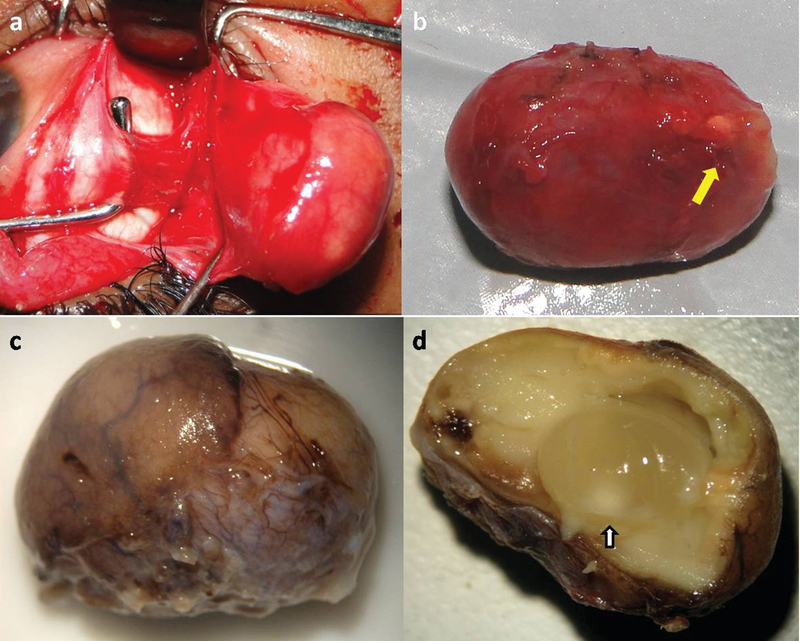
(a) Intraoperatively, the lateral rectus was isolated with the muscle hook for a better and minimally damaging dissection. (b) The in-to excised cystic mass with distinct anterior yellowish portion. (c) The gross specimen revealing the bilobed cystic mass with overlying blood vessels. The anterior brownish and posterior yellowish portions are clearly discernible. (d) An intact cyst with turbid contents showing a yellowish-white scolex (arrow). The surrounding region shows inflammatory fibrosis.

The B-scan ultrasonography of the right orbit revealed a highly echogenic structure within a hypoechoic cystic lesion inside the lateral rectus muscle belly [Figure 2(a)]. The axial scans of computed tomography (CT) showed a hyperdense spec inside a hypodense lesion over the anterolateral part of the right globe [Figure 2(b)] and a bulky lateral rectus belly containing another heterogenous lesion [Figure 2(c)]. The brain CT showed a healed granuloma in the left parietal lobe [Figure 2(d)]. The history, clinical features, and radiological features were suggestive of right twin-orbital myocysticercosis with healed neurocysticercosis. On retrospective history, there was a contributory history of frequent consumption of smoked pork. There was no history of headache,     vomiting, or seizure, and neurology clearance was obtained before prescribing oral albendazole (15 mg/kg/day in two divided doses) and oral steroids (1 mg/kg/day). The enzyme-linked immune sorbent assay (ELISA) for cysticercosis and stool examination was negative.

To prevent the spontaneous rupture and extrusion of the large, anterior, subconjunctival cyst, an “in-toto” surgical excision of the cyst was performed under general anesthesia. Intraoperatively, the lateral rectus muscle was hooked, and a gentle and blunt dissection was performed to remove the cyst in-toto [Figure 3(a)]. A reddish-yellow cystic mass (18 × 15 mm) was excised with an anteroinferior yellowish region suggestive of cysticercosis cyst [Figure 3(b)]. The formalin-fixed gross pathological specimen classically demonstrated a bilobed cystic mass with a smooth (orbital) surface and an intact pseudo capsule [Figure 3(c)]. The anterior part of superior calotte revealed an intact cyst with clear contents and pearly-white scolex [Figure 3(d)]. The posterior region showed collapsed cyst with surrounding inflammatory fibrosis. Oral albendazole was continued for six weeks with weekly tapering of oral steroids. At a 12 months follow-up, the child recovered completely without any recurrence or sequelae.

##  DISCUSSION

The infiltration of cysticerci (larvae of *Taenia solium*) into ophthalmic tissues is called ocular or adnexal cysticercosis. The larvae spread to ophthalmic tissues via the hematogenous route, and the involvement of extraocular muscles is known as orbital myocysticercosis.^[1]^ In adults, the orbital myocysticercosis may have an acute presentation akin to idiopathic orbital inflammatory disease (IOID), while in the younger age, it may present as an anteriorly prolapsed subconjunctival cyst.^[1,2,3]^ In the latter scenario, there can be another “twin-cyst” or a continuation of posterior cyst in the associated rectus belly.^[4]^ Orbital imaging (ultrasonography, CT, or MRI) may reveal the internal contents, extent, and association of cystic lesion inside the extraocular muscles or orbit.

The extraocular muscles have a rich vascular supply favoring the lodgment of cysticercosis larvae.^[1,2,3][4]^ Traditionally, medical management is the first line of treatment for orbital myocysticercosis, but an anterior subconjunctival cyst, amenable to surgical excision, can be removed, preventing spontaneous inflammatory rupture of the cyst. Acute painful orbital conditions should be included in the differential diagnosis of the orbital myocysticercosis. Our case depicts a classical presentation of orbital myocysticercosis with a radiologically detected posterior “twin-cyst” and its successful surgical management. The authors also want to emphasize on the therapeutic medical management of orbital myocysticercosis in case of posteriorly placed orbital cysts.

##  Financial Support and Sponsorship

Sri Kanchi Sankara Health & Educational Foundation, India.

##  Conflicts of Interest

There are no conflicts of interest.
